# Surgery for bilateral vocal fold paralysis: Systematic review and meta-analysis

**DOI:** 10.3389/fsurg.2022.956338

**Published:** 2022-07-22

**Authors:** Kai Titulaer, Peter Schlattmann, Orlando Guntinas-Lichius

**Affiliations:** ^1^ Department of Otorhinolaryngology, Jena University Hospital, Jena, Germany; ^2^ Department of Medical Statistics, Computer Sciences and Data Sciences, Jena University Hospital, Jena, Germany

**Keywords:** bilateral vocal fold paralysis, treatment outcome, meta-analysis, surgery, decannulation

## Abstract

**Objectives:**

To determine the decannulation rate (DR) and revision surgery rate after surgery for bilateral vocal fold paralysis (BVFP).

**Data Sources:**

Five databases (MEDLINE, PubMed, Embase, Web of Science, Scopus) were searched for the period 1908–2020.

**Methods:**

The systematic literature review followed the Preferred Reporting Items for Systematic Reviews and Meta-Analyses (PRISMA) guidelines. Data were pooled using a random-mixed-effects model. Randomized controlled trials and non-randomized studies (case-control, cohort, and case series) were included to assess DR and revision surgery rate after different surgical techniques for treatment of BVFP.

**Results:**

The search yielded 857 publications, of which 102 with 2802 patients were included. DR after different types of surgery was: arytenoid abduction (DR 0.93, 95%-confidence interval [CI], 0.86–0.97), endolaryngeal arytenoidectomy (DR 0.92, 95%-CI, 0.86–0.96), external arytenoidectomy (DR 0.94; 95%-CI, 0.71–0.99), external arytenoidectomy and lateralisation (DR 0.87; 95%-CI, 0.73–0.94), laterofixation (DR 0.95; 95%-CI, 0.91–0.97), posterior cordectomy (DR 0.97, 95%-CI, 0.94–0.99), posterior cordectomy and arytenoidectomy (DR 0.98, 95%-CI, 0.93–0.99), posterior cordectomy and subtotal arytenoidectomy (DR 0.98, 95%-CI, 0.88–1.00), posterior cordotomy (DR 0.96, 95%-CI, 0.84–0.99), reinnervation (0.69, 95%-CI, 0.12–0.97), subtotal arytenoidectomy (DR 1.00, 95%-CI, 0.00–1.00) and transverse cordotomy (DR 1.0, 95%-CI, 0.00–1.00). No significant difference between subgroups for DR could be found (Q = 15.67, df = 11, *p* = 0.1540). The between-study heterogeneity was low (*τ*2 = 2.2627; *τ* = 1.5042; I^2^ = 0.0%). Studies were at high risk of bias.

**Conclusion:**

BLVP is a rare disease and the study quality is insufficient. The existing studies suggest a publication bias and the literature review revealed that there is a lack of prospective controlled studies. There is a lack of standardized measures that takes into account both speech quality and respiratory function and allows adequate comparison of surgical methods.

## Introduction

Bilateral vocal fold paralysis (BVFP) is an uncommon condition in which patients are unable to abduct the vocal folds. This results in upper airway obstruction, usually manifested by variable degrees of stridor and/or dyspnoea of varied intensity, often requiring immediate surgical intervention (1). Some cases in which the symptoms worsened over a longer period of time requiring an intervention at a later time (2, 3).^.^ Most of the underlying lesions are iatrogenic damage to the peripheral recurrent laryngeal nerve due to neck surgery (thyroid, parathyroid glands, thymus, oesophagus, and carotid body paragangliomas) as well as cardiosurgical, thoracosurgical, and neurosurgical procedures. Thyroid surgery is the single most common cause of persistent iatrogenic bilateral cord paralysis and accounts for almost a quarter of all cases. The problem occurs in 1% of thyroidectomies (4, 5).

Until the late nineteenth century, tracheotomy was the only surgical method to treat dyspnoea resulting from the bilateral vocal fold paralysis (6). Since the mid-20th century, there have been surgical innovations, mainly minimally invasive endoscopic techniques (7). Recently, there have been experimental trials on reanimating the neurologically impaired larynx by reinnervation procedures or laryngeal pacing (8, 9).

The aim of this meta-analysis was to describe the variety of interventions aimed at restoring the airway patency in BVFP and to compare their success as measured by the decannulation rate (DR) and by the rate of revision surgery (RSR).

## Methods

### Literature search

Five electronic databases (MEDLINE, PubMed, Embase, Web of Science and Scopus) were screened with following Medical Subject Heading (MeSH) terms: “bilateral vocal fold paralysis”, “bilateral vocal fold palsy”, “BVFP”, “vocal cord paralysis” and “bilateral vocal cord immobility”. All studies published between January 1908 and December 2020 were considered. Moreover, reference lists of identified articles for additional relevant studies were hand-searched.

### Selection of cases

Two independent reviewers (K.T.; O.G.L.) reviewed abstracts and full texts. If they came to a different conclusion, a joint decision was made in a discussion. All studies were assessed against the following exclusion criteria: review articles, duplicate patients, absence of essential data (patient count, decannulation rate and operation type), multiple use of same patient dataset and animal studies.

### Data extraction

The following data were extracted from the included papers: number of patients, gender, mean age, publication type, intervention, decannulation rate (as primary outcome measure), time between diagnosis and therapy, duration of follow-up, rate of severe complications, rate of reoperation and Oxford Centre for Evidence-based Medicine (CEBM)-Score. Assuming that every patient requiring therapy for respiratory distress would receive a tracheostomy, the decannulation rate referred to all patients included in the respective study.

Subgroup analyses depended on the number of patients, so the therapy groups with less than three representatives had to be excluded from the meta-analysis and the remaining therapy approaches were only mentioned descriptively (Supplementary Table S1).

### Statistical analyses

The studies were categorised in subgroups by intervention type. Statistical analyses were carried out in R version 4.0.4 (10, 11). The meta package (version 4.18–0) was used to produce the pooled estimates and forest plots. The meta-analysis was conducted for the rate of decannulated patients (DR) and the revision surgery rate (RSR). Actually, these outcome parameters are proportions. Accordingly, parameter estimation was based on a logistic regression model with random effects fit by maximum likelihood (Laplace approximation). Separate estimates of the between-study heterogeneity were used to pool the results within subgroups of different surgical techniques. It was verified whether the parameter “surgical technique” used for grouping had an impact on both target variables (DR and RSR). Publication bias was assessed *via* Egger's test for funnel plot asymmetry. I² statistics were used to quantify statistical heterogeneity.

## Results

### Characteristics of the studies

A total of 837 titles were identified by searching the databases and journal that corresponded to the previously mentioned MeSH terms. Of these, 645 did not meet our study inclusion criteria. The remaining 192 articles were screened based on the review of their abstracts. This meant that a further 78 publications, including systematic reviews, animal studies and multiple publications on the same data set, had to be excluded from the study as they did not meet our inclusion criteria. Other reasons for exclusion were that the essential parameters such as the decannulation rate (DR) were not given or, in the case of comparative studies, these could not be assigned to a surgery type. After reviewing the full texts of the remaining papers, 102 articles were selected for meta-analysis (Supplementary Figure S1).

**Figure 1:**
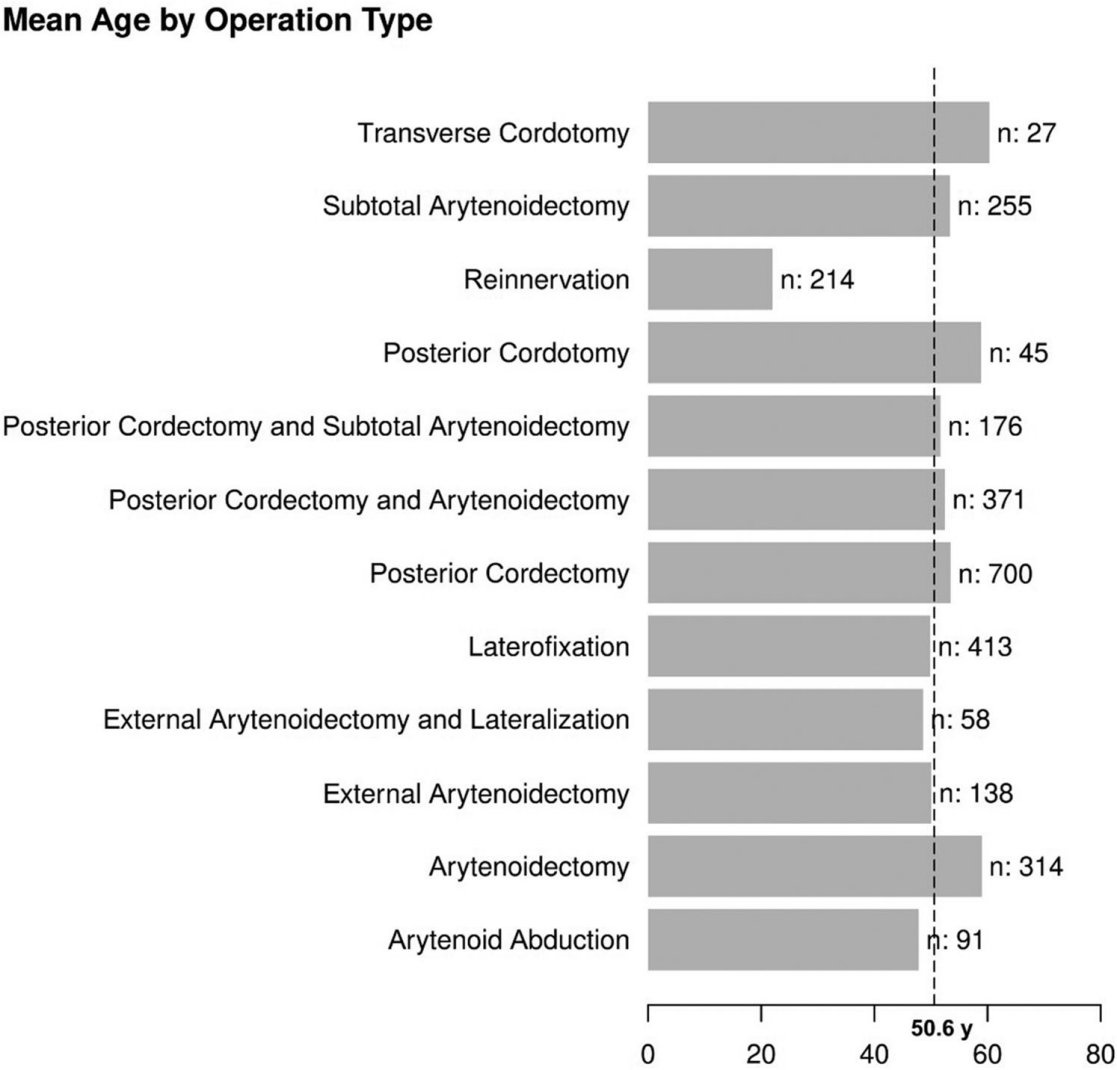
Mean age in years (y) of the patient in relation to chosen surgical technique. Dotted line shows weighted mean weight (total number of patients in brackets).

Table 1 displays the characteristics of 102 eligible studies, which were published between 1932 and 2019. Two studies were randomized controlled trials, 30 were prospective case series, 6 prospective cohort studies, 61 retrospective case series and 5 retrospective cohort studies. Sample sizes ranged from 1 to 202, with a total of 2802 patients evaluated across all studies with a weighted mean age of 50.6 years (Figure 1). In the random effects subgroup (surgery type) analysis, we could not find any significant differences with regard to the parameter decannulation rate (Q = 15.67, *p* = 0.1540). The heterogeneity was low (*τ*^2^ = 2.2627; *τ* = 1.5042; I^2^ = 0.0%).

**Table 1:** List of included studies.

Study	Type	Patients^a^	Surgical technique	Mean age, years	Diagnose / Intervention-Interval, years	Follow up, years, mean ± SD	SC, %	Revisions, %	CEBM	Decannulation Rate, %
Al-Fattah et al. (12) (2006)	PCaS	45 (7 males)	Posterior Cordectomy and Subtotal Arytenoidectomy	48	–	–	0	7	4	100
Amedee and Mann (13) (1989)	PCaS	18 (3 males)	Laterofixation	–	–	–	11	0	4	100
Anand and Galantich (14) (1990)	RCaS	10	Posterior Cordectomy and Subtotal Arytenoidectomy	–	–	–	0	0	4	100
Anand et al. (15) (2015)	RCoS	31, 13	Posterior Cordectomy, External Arytenoidectomy and Lateralization	–, –	–, –	–, –	–, –	13, 23	4	90, 77
										
Asik et al. (16) (2016)	PCaS	11 (2 males)	Posterior Cordectomy	46.6	4.04	0.17	0	0	4	100
Bajaj et al. (17) (2009)	RCaS	9 (3 males)	Transverse Cordotomy	–	2	2.33	0	0	4	100
Benninger et al. (18) (2018)	RCaS	15 (4 males)	Posterior Cordotomy	57	–	1.41	13	47	4	93
Berendes (19) (1949)	RCaS	3 (1 male)	Laterofixation	44.7	–		33	33	4	67
Bernstein et al. (20) (2012)	RCaS	8	Posterior Cordotomy	62.3	–	1.32 ± 1.03	0	0	4	88
Bigenzahn and Höfler (21) (1995)	RCaS	84 (17 males)	Posterior Cordectomy	60.2	–	0.67	0	21	4	100
Bizakis et al. (22) (2004)	PCaS	18	Posterior Cordectomy and Arytenoidectomy	53.1	1.53 ± 1.79	–	0	0	4	100
Bosley et al. (23) (2005)	RCoS	6 (3 males), 11 (6 males)	Subtotal Arytenoidectomy, Transverse Cordotomy	52.5, 56.9	–, –	2.47 ± 1.69, 2.80 ± 1.72	17, 18	17, 9	4	100, 100
Brake and Anderson (24) (2015)	RCaS	21	Posterior Cordectomy and Arytenoidectomy	–	–	–	10	33	4	95
Carlens (25) (1954)	RCaS	22	External Arytenoidectomy and Lateralization	–	–	–	–	45	4	91
Clerf (26) (1950)	RCaS	66 (10 males)	Arytenoid Abduction	–	–	–	2	6	4	95
Crumley (27) (1983)	RCaS	4 (3 males)	Reinnervation	–	5.5 ± 6.34	2.5 ± 1.73	25	0	4	75
Crumley (28) (1993)	RCaS	8	Subtotal Arytenoidectomy	–	–	–	0	0	4	50
Danino et al. (29) (2000)	RCaS	6 (2 males)	External Arytenoidectomy	–	–	–	–	–	4	100
de Bord (30) (1953)	RCaS	4 (0 males)	External Arytenoidectomy	^46^	5.27 ± 5.31	1.29 ± 0.89	0	0	4	100
de Campora et al. (31) (1985)	RCaS	12	Arytenoidectomy	–	–	2	–	0	4	100
Dennis and Kashima (32) (1989)	RCaS	6 (0 males)	Posterior Cordectomy	47.3	17.75 ± 14.01	3.82 ± 1.76	17	33	4	100
Dispenza et al. (33) (2012)	RCoS	5, 25	Arytenoidectomy, Posterior Cordectomy and Arytenoidectomy	–, –	–, –	–, –	–, –	20 4	4	60, 96
Doyle et al. (34) (1967)	RCaS	2 (1 male)	Reinnervation	22	0	0.58 ± 0.35	0	0	4	100
Dursun and Gökcan (35) (2006)	PCaS	22 (8 males)	Posterior Cordectomy	45	–	2.77	32	27	4	91
Eckel and Vössing (36) (1996)	PCoS	5	Posterior Cordectomy	–	–	–	0	20	3b	100
Eckel et al. (37) (1994)	PCoS	18 (5 males), 10 (2 males)	Posterior Cordectomy, Arytenoidectomy	56, 59	0.58, 0.92	1.42, 1.08	6, 0	6, 0	3b	94, 90
Eckel (38) (1991)	RCaS	15 (4 males)	Posterior Cordectomy	–	–	–	7	7	4	93
Ejnell et al. (39) (1984)	PCaS	13 (3 males)	Laterofixation	55.2	15	0.84 ± 0.73	–	39	4	92
Ejnell and Tisell (40) (1993)	RCaS	4 (1 male)	Laterofixation	51.3	0	0.17 ± 0.05	0	0	4	100
Ezzat et al. (41) (2010)	PCaS	21 (8 males)	Laterofixation	36	–	0.5	10	19	4	95
Gammert (42) (1977)	RCaS	14	Posterior Cordectomy and Subtotal Arytenoidectomy	–	–	2	7	0	4	86
Geterud et al. (43) (1990)	RCaS	11 (4 males)	Laterofixation	65	–	7.3	0	27	4	100
Gorphe et al. (44) (2013)	PCaS	20 (7 males)	Subtotal Arytenoidectomy	52	–	0.25	5	0	4	100
Gupta et al. (45) (1997)	RCaS	5	Posterior Cordectomy	–	–	–	–	–	4	60
Hans (46) (2000)	PCaS	4 (2 males)	Posterior Cordectomy	68	–	2	0	0	4	100
Harnisch et al. (47) (2008)	PCaS	2 (1 male), 1 (0 male), 7 (1 male)	Posterior Cordectomy, Laterofixation, Transverse Cordotomy	67, 71, 65.7	–, –, –	2.67 ± 1.18, 4.67, 1.81 ± 1.63	50, 100, 43	50, 100, 43	4	100, 100, 100
Havens (48) (1953)	RCaS	50	Arytenoidectomy	–	–	–	–	14	4	96
Helmus (49) (1972)	RCaS	10 (1 male)	External Arytenoidectomy	53.1	9.08 ± 6.84		40	0	4	100
Herberhold and Hück (50) (1995)	RCaS	22	Posterior Cordotomy	–	–	–	–	–	4	100
Holm et al .(51) (1989)	PCaS	24 (12 males)	Posterior Cordectomy and Arytenoidectomy	58	9	3.25 ± 2.39	13	42	4	79
Hoover (52) (1932)	RCaS	4 (0 male)	Subtotal Arytenoidectomy	38.8	1.38 ± 0.44	–	25	50	4	100
Issac (53) (2017)	PCaS	31 (3 males)	Posterior Cordectomy	46.7	–	–	10	0	4	100
Jackowska et al. (4) (2018)	PCaS	132 (11 males)	Posterior Cordectomy	63	4.92	–	–	43	4	89
Jóri et al. (54) (1998)	RCaS	4 (1 male)	Laterofixation	51	0.02 ± 0.02	0.81 ± 0.38	25	25	4	100
Joshua et al. (55) (2004)	RCaS	10 (5 males)	Posterior Cordectomy	61	–	–	30	0	4	100
Kelly (56) (1941)	RCaS	2 (1 male)	Arytenoidectomy	–	–	–	–	–	4	100
Khalifa (57) (2005)	PCaS	22 (8 males)	Posterior Cordectomy	–	–	–	18	18	4	82
Khalil and Abdel Tawab (58) (2014)	PCaS	18 (8 males)	Posterior Cordectomy	47.4	–	1	17	11	4	100
King (59) (1939)	RCaS	3 (0 males)	Arytenoid Abduction	59.3	8.67 ± 3.21	–	0	0	4	67
Kleinsasser and Nolte (60) (1981)	RCaS	110 (5 males)	Posterior Cordectomy and Arytenoidectomy	53.2	–	–	5	1	4	96
Korkmaz et al. (61) (2015)	RCaS	47 (3 males)	Laterofixation	50.6	4.6	1.57	6	–	4	100
Kressner (62) (1949)	RCaS	57	Laterofixation	–	–	–	–	–	4	88
Laccourreye et al. (63) (1999)	RCaS	25 (6 males)	Posterior Cordectomy	63	–	3.76 ± 2.87	0	32	4	100
Lagier et al. (64) (2009)	RCaS	11	Posterior Cordectomy	0.8	0.67	2.25	36	–	4	91
Lawson et al. (65) (1996)	PCoS	37 (17 males), 9 (1 male)	Posterior Cordectomy, Subtotal Arytenoidectomy	57, 62	–, –	–, –	0, 11	0, 11	4	100, 100
León et al. (66) (2001)	RCaS	21	Arytenoidectomy	–	–	–	–	–	4	86
Lidia et al. (67) (2010)	RCaS	10	Laterofixation	–	–	–	–	50	4	80
Lim (68) (1985)	RCaS	20 (6 males)	Arytenoidectomy	–	–	–	5	0	4	100
Luczaj et al. (69) (2008)	PCaS	36 (7 males)	Posterior Cordectomy	42	–	–	0	17	4	92
Manolopoulos et al. (70) (1999)	RCaS	18 (4 males)	Posterior Cordectomy	41.4	1.21 ± 0.62	–	50	50	4	89
Maurizi et al. (71) (1999)	PCaS	39 (8 males)	Posterior Cordectomy and Subtotal Arytenoidectomy	–	–	–	13	13	4	100
Meurman (72) (1943)	RCaS	8 (2 males)	Arytenoid Abduction	32.9	6.59 ± 7.52	4.05 ± 4.22	38	13	4	88
Misiołek et al. (73) (2012)	PCoS	36 (3 males), 21 (5 males)	Posterior Cordectomy and Arytenoidectomy, Laterofixation	52.0, 54.4	–	–	0, 0	0, 0	3b	97, 100
Misiołek et al. (74) (2007)	PCaS	30 (6 males)	Posterior Cordectomy and Arytenoidectomy	58.5	–	5	0	0	4	100
Mohammed et al. (75) (2013)	RCT	10 (4 males) 10 (0 males)	Posterior Cordectomy, Posterior Cordectomy	53.5, 51.1	–	–, –	0, 100	0, 20	1b	100, 100
Mondal et al. (76) (2005)	RCaS	8	Arytenoid Abduction	–	–	–	0	0	4	100
Motta et al. (77) (2003)	RCoS	83 (28 males)	Posterior Cordectomy and Arytenoidectomy	48	–	10	13	10	4	100
Moustafa et al. (78) (1992)	PCoS	10, 15, 11	External Arytenoidectomy, Arytenoidectomy, Laterofixation	–, –, –	–, –, –	–, –, –	–, –, –	–, –, 9	4	30, 73, 91
Nawka et al. (79) (2015)	PCoS	3, 4, 23	Posterior Cordectomy, Laterofixation, Posterior Cordectomy and Subtotal Arytenoidectomy	–, –, –	–, –, –	–, –, –	–, –, –	–, –, –	3b	100, 100, 100
Newman and Work (80) (1976)	PCaS	21 (1 male)	External Arytenoidectomy	–	–	–	–	24	4	76
Olthoff et al. (81) (2005)	PCaS	17 (4 males)	Posterior Cordectomy	58	4	0.92	0	24	4	100
Ossoff et al. (82) (1984)	RCaS	11 (1 male)	Arytenoidectomy	––	–	–	9	0	4	91
Özdemir et al. (83) (2013)	RCaS	66 (8 males)	Posterior Cordectomy	48	–	3.33	12	18	4	100
Pearlman and Killian (84) (1953)	RCaS	4 (2 males)	External Arytenoidectomy	47	8.56 ± 11.03	0.15 ± 0.23	0	0	4	100
Pinto et al. (85) (2011)	RCaS	18 (4 males)	Subtotal Arytenoidectomy	45	–	–	11	11	4	100
Plouin-Gaudon et al. (86) (2005)	RCaS	69 (35 males)	Subtotal Arytenoidectomy	56	–	4.17 ± 3.67	3	0	4	100
Prasad (87) (1985)	RCaS	6 (0 males)	Subtotal Arytenoidectomy	–	–	0.4 ± 0.2	0	0	4	100
Rao et al. (88) (2015)	RCoS	10, 15	Posterior Cordectomy, Laterofixation	–, –	–, –	0.25, 0.52	20, 73	–, –	3b	100, 93
Remacle et al. (89) (1996)	PCaS	41 (16 males)	Subtotal Arytenoidectomy	55	–	4.67 ± 2.42	2	0	4	100
Rinne (90) (1991)	RCaS	34 (10 males)	Laterofixation	54.3	9.75	15.83	–	38	4	79
Rontal and Rontal (91) (1994)	RCaS	8 (5 males)	Posterior Cordectomy and Subtotal Arytenoidectomy	–	–	1	0	13	4	100
Rovó et al. (92) (2001)	PCaS	25 (5 males)	Laterofixation	–	0.06	–	16	0	4	96
Sato et al. (93) (2001)	RCaS	9	Arytenoidectomy	–	–	–	–	–	4	100
Scheer (94) (1953)	RCaS	1 (1 male)	Arytenoidectomy	64	1.5	0.08	0	100	4	100
Segas et al. (95) (2001)	PCaS	20 (8 males)	Posterior Cordectomy	49.8	1.1 ± 0.62	–	45	45	4	90
Sessions et al. (96) (1976)	PCaS	55 (21 males)	External Arytenoidectomy	50	–	–	–	38	4	91
Sethi et al. (97) (2016)	RCaS	14	Posterior Cordectomy	48.6	–	0.5	–	0	4	100
Shvero et al. (98) (2003)	RCaS	22 (15 males)	Posterior Cordectomy and Subtotal Arytenoidectomy	55.6	2.2 ± 1.03	–	27	5	4	91
Songu et al. (99) (2013)	RCaS	17 (0 males)	Laterofixation	48.4	2.14 ± 1.41	1.98 ± 1	0	6	4	100
Su et al. (100) (2014)	RCaS	20 (3 males)	Laterofixation	53.2	2.68 ± 1.8	2.75 ± 1.33	15	20	4	95
Thornell (101) (1948)	RCaS	1 (0 males)	Arytenoidectomy	54	2.67	0.15	0	0	4	100
Tucker (102) (1989)	RCaS	202	Reinnervation	–	–	2	2	26	4	89
Virmani and Dabholkar (103) (2016)	PCaS	7 (2 males)	Posterior Cordectomy	36.1	7.79 ± 6.61	0.5	0	0	4	100
Werner and Lippert (104) (2002)	PCaS	40 (3 males)	Laterofixation	42.9	–	1.46	0	0	4	98
Whicker and Devine (105) (1972)	RCaS	147	Arytenoidectomy	–	11.46	11	5	27	4	92
Wigand et al. (106) (1969)	RCaS	6	Reinnervation	–	–	0.42	–	–	4	0
Woodman (107) (1946)	RCaS	1 (1 male)	External Arytenoidectomy and Lateralization	45	5	0.67	0	0	4	100
Woodman (108) (1949)	RCaS	24	External Arytenoidectomy	–	–	–	–	–	4	96
Woodson and Weiss (109) (2007)	PCaS	6 (3 males)	Arytenoid Abduction	62	–	–	–	–	4	83
Yilmaz (8) (2019)	PCaS	64 (8 males)	Subtotal Arytenoidectomy	52	–	2	2	13	4	100
Yilmaz et al. (110) (2013)	RCT	10, 10	Arytenoidectomy, Subtotal Arytenoidectomy	–, –	–, –	–, –	–, –	–, –	2b	100, 100
Zenev and Sapundzhiev (111) (2015)	PCaS	4 (1 male)	External Arytenoidectomy	50.5	–	1.38 ± 0.77	25	0	4	100

SC, severe complications; RCT, prospective randomized controlled study; RCaS, retrospective case series; RCoS, retrospective cohort study; PCaS, prospective case series; PCoS, prospective cohort study; CEBM, oxford center of evidence based medicine score; SD, standard deviation.

^a^ No differentiation between female and male patients.

### Risk of bias assessment

**Figure 2:**
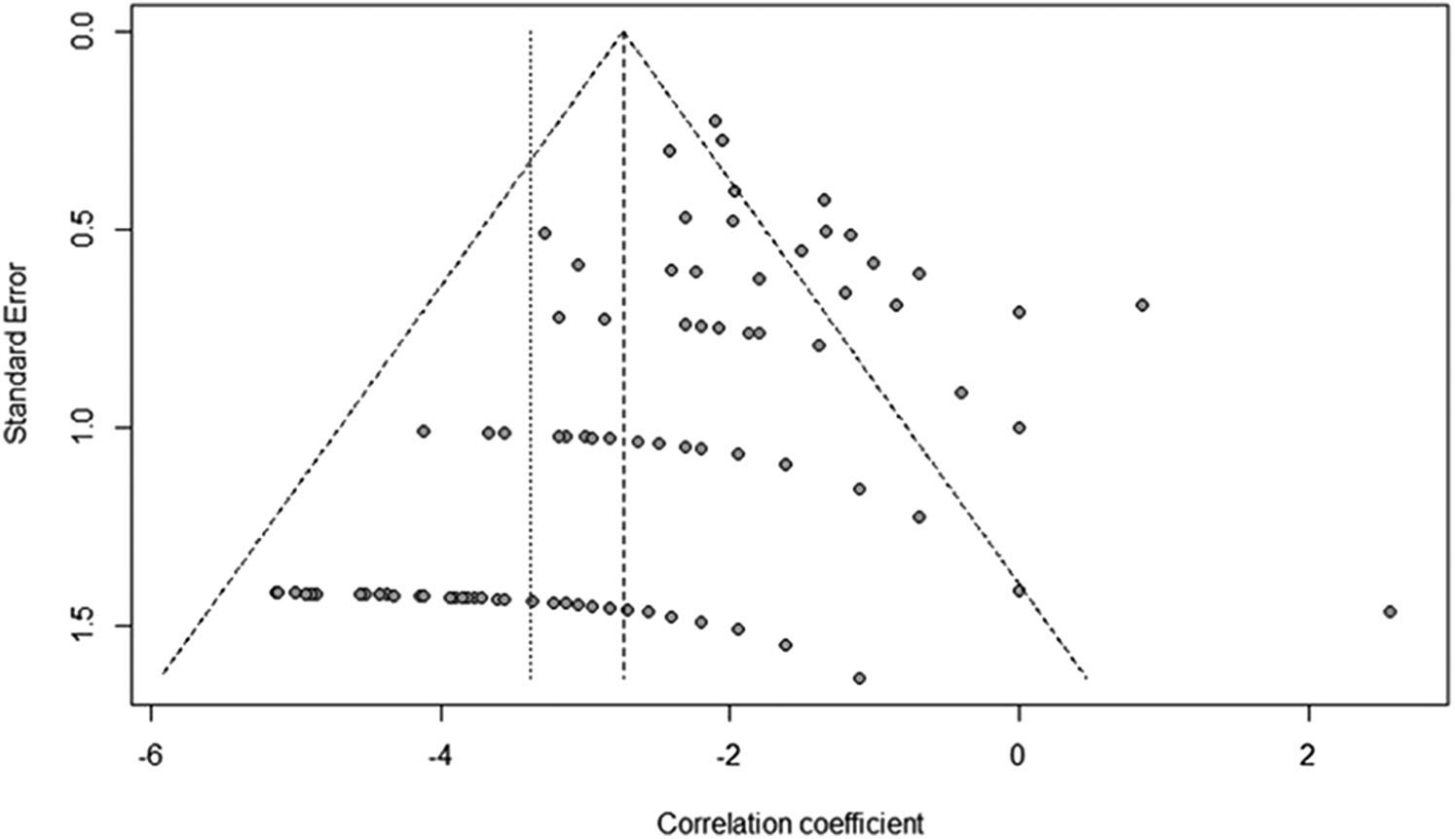
Publication bias: the asymmetric funnel plot makes a publication bias likely.

The data was visualized with a funnel plot (Figure 2). Because of result of the Egger's test (t = 9.26, *p* < 0.0001), a bias was suspected, in particular a publication bias due to the predominant study type (retrospective case series).

### Association of surgical techniques to decannulation rate

The decannulation rate varied from 50% to 100% between the studies. No significant difference between surgical techniques could be determined with regards for DR (Q = 15.67, df = 11, *p* = 0.1540).

**Figure 3:**
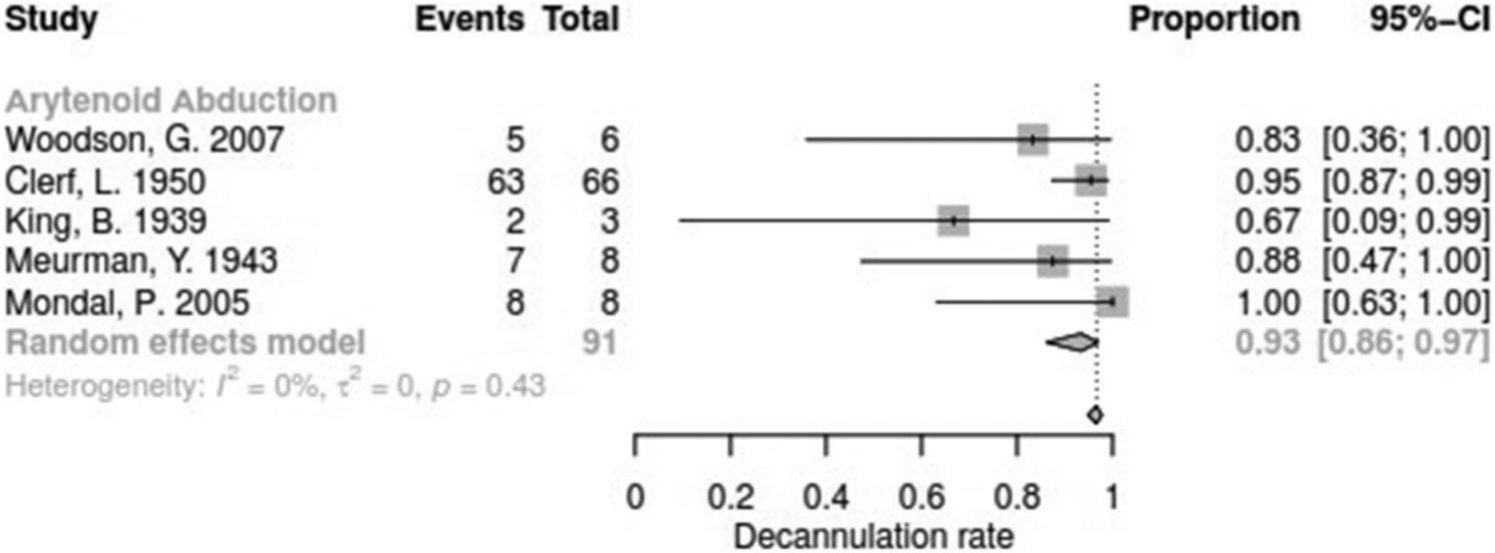
Forest plot of decannulation rates in studies on on arytenoid abduction.

**Figure 4:**
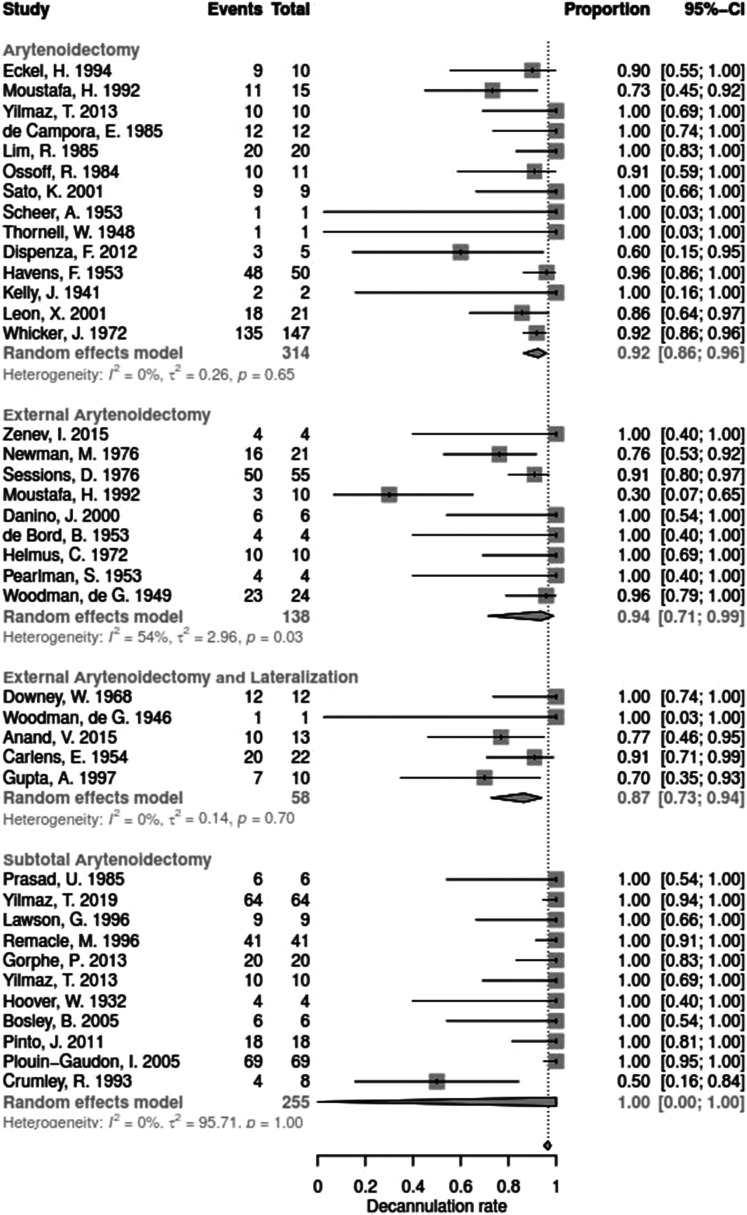
Forest plot of decannulation rates in studies on arytenoidectomy.

**Figure 5:**
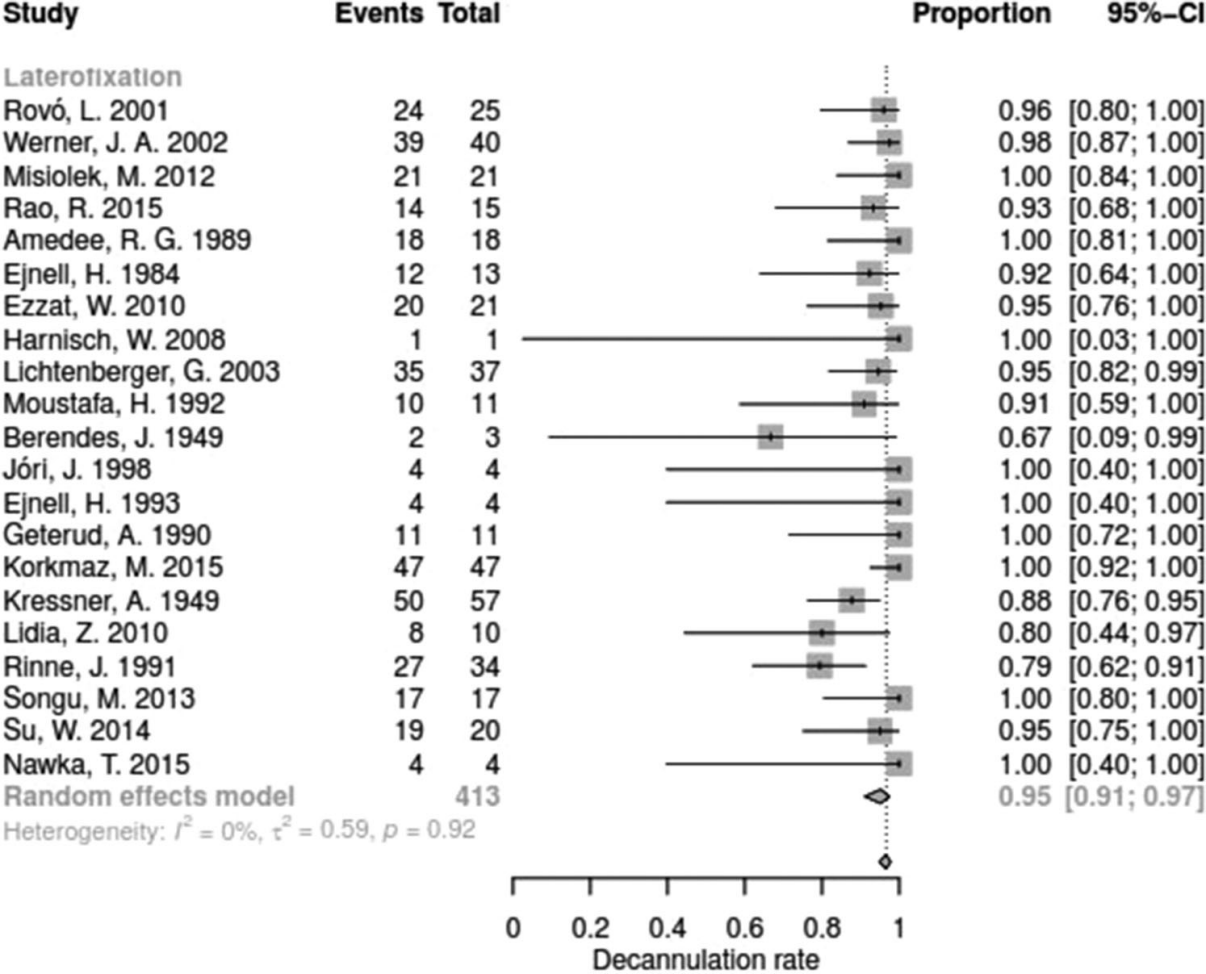
Forest plot of decannulation rates in studies on laterofixation.

**Figure 6:**
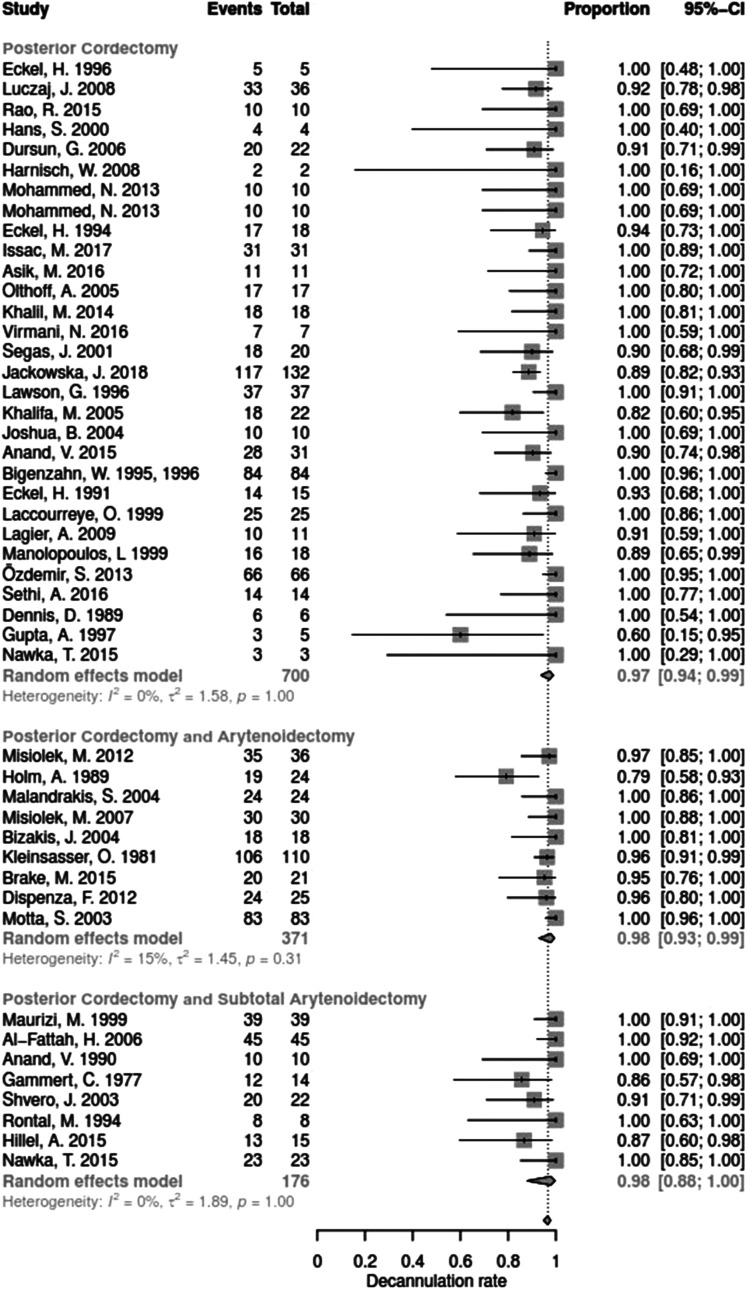
Forest plot of decannulation rates in studies on cordectomy.

**Figure 7:**
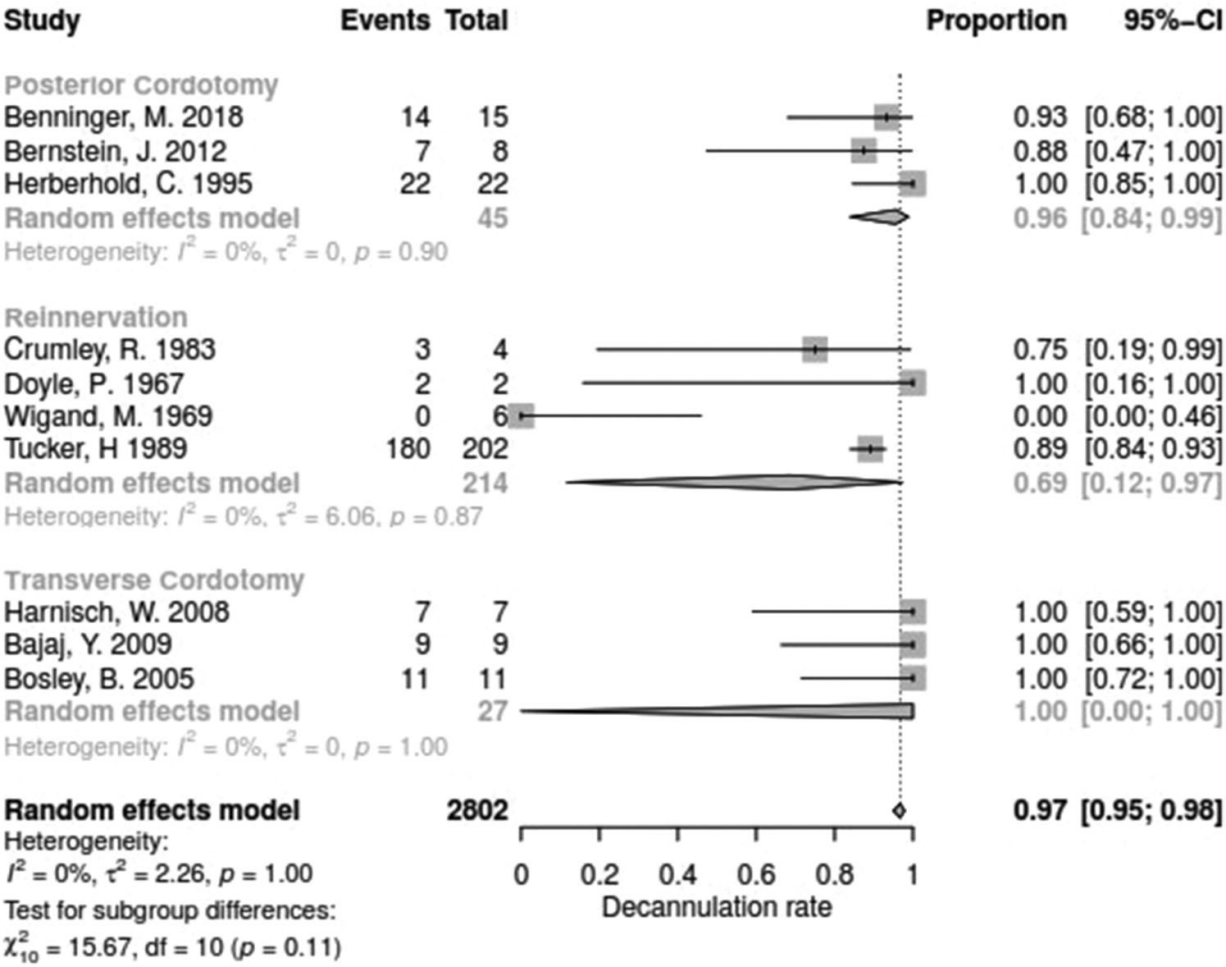
Forest plot of decannulation rates in studies on cordotomy or on reinnervation surgery.

Data for arytenoid abduction was available in five studies with a total of 91 patients (DR = 0.93; 95%-CI, 0.86–0.97, Figure 3). Fourteen studies reported on 314 patients treated by arytenoidectomy (DR = 0.92; 95%-CI, 0.86–0.96) and 11 studies on 255 patients treated by subtotal arytenoidectomy (DR = 1.0; 95%-CI, 0.00–1.00). Nine studies reported on 138 patients treated by external arytenoidectomy (DR = 0.94; 95%-CI, 0.71–0.99) and five studies reported on 58 patients treated by external arytenoidectomy and additional lateralization (DR = 0.87; 95%-CI, 0.73–0.94) (Figure 4). Laterofixation was reported in 21 studies involving 413 patients (DR = 0.95; 95%-CI, 0.91–0.97, Figure 5). Seven hundred (700) patients who underwent posterior cordectomy were reported in 30 studies (DR = 0.97; 95%-CI, 0.94–0.99). In 371 patients reported in nine studies, a arytenoidectomy was also performed (DR = 0.98; 95%-CI, 0.93–0.99). Additional subtotal arytenoidectomy was performed instead in 176 patients reported in eight studies (DR = 0.98; 95%-CI, 0.88–1.00) (Figure 6). Posterior cordotomy was described in three studies involving a total of 45 patients (DR = 0.96; 95%-CI, 0.84–0.99, Figure 7). Transverse cordotomy was the intervention in three studies with a total of 27 patients (DR = 1.00; 95%-CI, 0.00–1.00). In the reinnervation group, there was the lowest decannulation rate (DR = 0.69; 95%-CI, 0.12–0.97, Figure 7). Patients receiving reinnervation surgery were younger than patients receiving other types of surgery (cf. Figure 1).

### Association of surgical technique to revision surgery rate

The revision surgery rate (RSR) varied from 0% to 100% between the studies. The difference between surgical techniques in terms of RSR was significant (Q = 38.58, df = 10, *p* < 0.0001). In 98 studies, RSR was reported for 2453 patients. The heterogeneity was low (*τ*^2^ = 1.9139; *τ* = 1.3834; I^2^ = 32.8%)

RSR for arytenoid abduction was reported in four studies and 85 patients (RSR = 0.06; 95%-CI, 0.02–0.13; Supplementary Figure S2). The 257 patients in the subgroup arytenoidectomy included nine studies with a pooled RSR of 0.06 (95%-CI, 0.01–0.28). Furthermore, the external arytenoidectomy was described in six studies (98 patients; RSR = 0.08; 95%-CI, 0.01–0.53). In combination with lateralization, the external arytenoidectomy was mentioned in four studies (48 patients; RSR = 0.10; 95%-CI, 0.04–0.23; Supplementary Figure S3). Laterofixation had a pooled revision rate of 0.11 (RSR = 0.11; 95%-CI, 0.04–0.23) for 290 patients in 17 studies (Supplementary Figure S4).

Twenty-six (112) studies have reported on RSRs in posterior cordectomy (RSR = 0.13; 95%-CI, 0.08–0.21) in 671 patients. A further nine studies combined this with arytenoidectomy (371 patients; RSR = 0.02; 95%-CI, 0.00–0.14) and another seven studies with subtotal arytenoidectomy (153 patients; revision rate 0.09; 95%-CI, 0.05–0.14: Supplementary Figure S5). The RSRs for subtotal arytenoidectomy alone have been described in 10 studies (RSR = 0.03; 95%-CI, 0.01–0.15) in 245 patients.

For the reinnervation subgroup (208 patients; RSR = 0.25; 95%-CI, 0.20–0.32) and the transverse cordotomy group (27 patients; RSR = 0.12; 95%-CI, 0.02–0.48), there were only three studies each that included statements about the RSRs (Supplementary Figure S6).

## Discussion

All surgical techniques investigated in this meta-analysis, except for the reinnervation techniques, showed high DR (87%–100%). No significant difference was found between the techniques in terms of DR. Since progressive atrophy of the laryngeal muscles is likely if the denervation is permanent and ankyloses of the non-moving cricoarytenoid joint is possible, timely treatment is essential. In both Tucker's and Wigand's techniques of recurrent nerve reinnervation, the failures were mostly due to already atrophic muscles or ankyloses of the cricoarytenoid joint so that no lasting success could be (102, 106). Here, it is necessary to weigh up the possibility of a spontaneous remission in the first 6 months. Due to the assumed publication bias, an overestimation of the positive results regarding DR should be considered.

The RSR showed a wider spread of efficacy (revision rate 0%–100%). The transverse cordotomy technique showed the lowest RSR of 1%. The reinnervation technique had the highest RSR of 25%, whereby account must be taken that the group of patients who underwent reinnervation surgery had the lowest mean age with 22 years. A possible explanation for the higher revision rate in younger patients treated with reinnervation could be increased activity, which is associated with a higher demand for respiratory function. The reinnervation techniques became more sophisticated in the recent years (113). More publications with larger case numbers achieving even better results can be expected in the future. Concerning the transverse cordotomy technique, it has to be taken into account that this is a bilateral procedure, so a larger glottal gap and consequently easier breathing is likely, which, however, has a disadvantageous effect on speech, but this was not investigated here.

Various explanations for variability of RSR could be found. On the one hand, all surgical techniques can lead to very variable degree of granuloma and oedema formation. Larger glottis gaps may increase the risk of aspiration (6). In addition, the surgeon must find in each individual case a compromise between sufficient airflow and speech preservation, which may justify a cautious approach to surgeries with as less resection extent as possible (114), and thereby higher risk of revision surgery. Some variability of the outcome within the same surgical technique can be explained by the learning curve of the individual surgeon or by the different experience within the involved head and neck surgeons. It has also be taken into account, that some of the analysed techniques were easier to perform that other ones. In summary, the inter-individual variability of the outcome of each technique seems to be higher than the variability between different surgical techniques. For the evaluation of the success of a surgical technique, it is also important that a sufficiently long follow-up period after surgery is given, as scar and granulation tissue can subsequently cause deterioration in breathing. This factors could not be investigated further in this meta-analysis, as most publications did not specify the follow-up period. For those who provided information, their mean value varied between 0.08 and 15.83 years (cf. Table 1).

A meta-analysis by Thorpe and Kanotra examined only paediatric patients (115). They compared the decannulation rates of four different surgical techniques (suture lateralization, cricoid split, arytenoidectomy and cordectomy/cordotomy). In addition, no difference in decannulation rates was found between the surgical techniques, but glottic widening surgery after tracheostomy was found to increase the decannulation rate. A meta-analysis on types and timing of surgery for BVFP after thyroidectomy revealed that outcome as more variable after bilateral posterior cordectomy than after early laterofixation and combined laser arytenoidectomy with posterior cordectomy after 12 months (116). Unfortunately, the definition of the outcome criteria remains unclear in this study. Furthermore, there were systematic reviews that attempted to collect the multitude of surgical procedures and provide an overview and decision support (1, 6, 27, 117). A superior surgical technique could not be identified, so it was advised to choose a surgical technique within the expertise of the surgeon and a method that is adapted to the patient's wishes and needs (e.g. higher risk of aspiration in arytenoidectomy) (1, 117). Eckel in his systematic review also chose the decannulation rate as a measure of respiratory disability (with DR of 100%–69.4%) (1).

As long as approaches to restore vocal cord function in permanent BVLP using reinnervation (34, 106, 118), or laryngeal pacemakers (119–121), only have been tried in very small clinical trials, it remains open if these techniques can find the right balance between restoring the airway and maintaining an adequate voice (110). For example, a large glottis gap in the mobile part of the vocal folds leads to impaired voice, but a glottis gap that is too small leads to respiratory distress^7^ and makes a decannulation impossible. An unlikely functionally complete reinnervation after recurrent nerve reconstruction surgery could only be expected if the original nerve fibres responsible for adduction and abduction, respectively, are reconnected correctly to the respective original target muscles (1).

BVFP is a rare disease and as a result, the published clinical case series often have too few cases to draw statistically significant conclusions (7). The heterogeneity among the chosen clinical parameters to determine the success of a therapy (e.g. forced expiratory pressure in 1 s [FEV1] or maximum phonation time) complicated the comparison. The most common detectable parameter was the DR. In the selected 102 studies, only 13% reported on FEV1, 17% on maximum phonation time and 9% on jitter/shimmer. That is why DR was chosen as a parameter to measure success in this meta-analysis, even though it only examines part of the problem (airway). This strategy provided only limited information about the extent to which respiratory function has been restored (e.g. whether sport is possible or only everyday activities) and no information about vocal function.

Although all relevant studies were included, a risk of publication bias could not be excluded, which was unavoidable due to the rarity of the disease and a lack of high-quality studies (the predominant study type is retrospective case series).

BVFP often already occurs due to neurapraxia of the recurrent laryngeal nerve, so that regeneration is possible (112). Up to which time this can still take place is not fully clarified. A period between 6 and 12 months is discussed (6, 112, 114, 122, 123). Only after this period a permanent paralysis of the vocal folds can be assumed. The mean time between diagnosis and intervention varied in this meta-analysis between 0.08 and 17.75 years (cf. Table 1). In most cases, a unilateral improvement in the function of the vocal cords would be sufficient (92, 104). In unilateral interventions, it cannot be ruled out with certainty that the success is not due to a spontaneous remission of the opposite side without further diagnostics in the period up to 12 months. However, atrophy can also occur within 12 months, jeopardising the success of, for example, reinnervation.

## Conclusions

In conclusion, no significant difference in decannulation rates was found between the surgical techniques studied. Since the first clinical experiments around 1908 by Citelli (124), more than 100 years of research have passed and no common therapy standard and measurement of success has been established. There is an urgent need for prospective, randomised clinical trials and the definition of parameters for an objective evaluation of the success of therapy beyond decannulation and revision surgery rate in terms of voice quality, swallowing function, and adequate airway. This applies to patients who require therapy immediately after first occurrence of BLVP as well as to patients with increasing symptoms in the later time course of the disease.

## Data Availability

The raw data supporting the conclusions of this article will be made available by the authors, without undue reservation.
