# Supplementary material for: Surgery for bilateral vocal fold paralysis: Systematic review and meta-analysis
Source: Front Surg. 2022 Jul 22;9:956338. doi: 10.3389/fsurg.2022.956338 (PMC9354550; doi:10.3389/fsurg.2022.956338)
Supplement: Supplementary file 1 [file Data_Sheet_2_v1.docx]

**Supplement Table 1**

**Surgery for Bilateral Vocal Fold Paralysis: Systematic Review and Meta-Analysis**

Kai Titulaer^1^, Peter Schlattmann, MD PhD^2^ Orlando Guntinas-Lichius, MD^1*^

^1^Department of Otorhinolaryngology, Jena University Hospital, Jena, Germany

^2^Department of Medical Statistics, Computer Sciences and Data Sciences, Jena University Hospital, Jena, Germany

| Supplement Table 1: List of excluded studies. | | | | | | | | | | |
| --- | --- | --- | --- | --- | --- | --- | --- | --- | --- | --- |
| Study | Type | Patients* | Surgical technique | Mean age, years | Diagnosis / Intervention-Interval,  years | Follow up, years, mean ± SD | SC, % | Revisions, % | CEBM | Decannulation Rate, % |
| Anand et al. (1)  (2015) | RCoS | 10 | Cordoplasty | - | - | - | 0 | 0 | 4 | 100 |
| Bahre (2)  (1930) | RCaS | 2 (1 male) | Vocal Cord Dislocation | 43 | - | - | 0 | 0 | 4 | 100 |
| Billante et al. (3)  (2002) | RCaS | 2 (0 males) | Laryngeal Pacemaker and Botulinum-Toxin Injection | - | - | 1.67 ± 1.41 | 0 | 0 | 4 | 50 |
| Gadkaree et al. (4)  (2018) | RCoS | 4,  2 | Cordectomy,  Open Framework Surgery | -,  - | -,  - | -,  - | -,  - | 50,  0 | 4 | 50,  100 |
| Gupta et al. (5)  (1997) | RCaS | 12 | Arytenoidectomy and Laterofixation | - | - | - | - | - | 4 | 67 |
| Jackson (6)  (1922) | RCaS | 7 (2 males) | Posterior Ventriculo-Cordectomy | 32.6 | 1.94 ± 0.71 | 1.71 ± 1.50 | 0 | 0 | 4 | 100 |
| Langnickel (7)  (1976) | PCaS | 62 | Vertico-Lateral Elevation | - | - | 1 | 2 | 2 | 4 | 100 |
| Müller (8)  (2017) | PCaS | 7 (2 males) | Laryngeal Pacemaker | 42.3 | 12.8 ± 8.0 | 0.5 | 0 | 29 | 4 | 100 |
| Nawka et al. (9)  (2015) | PCoS | 2,  2 | Posterior Cordectomy and Laterofixation,  Subtotal Arytenoidectomy and Laterofixation | -,  - | -,  - | -,  - | -,  - | -,  - | 3b | 100,  100 |
| Pia et al. (10)  (1999) | PCaS | 41 (8 males) | Posterior Ventriculo-Cordectomy | 55.4 | - | 3.58 | 0 | 12 | 4 | 88 |
| Ptok and Schönweiler (11)  (2001) | RCaS | 1 (0 males) | Botulinum-Toxin Injection | 80 | 4 | 0.33 | 0 | 0 | 4 | 100 |
| Remsen et al. (12)  (1985) | RCaS | 11 (3 males) | Arytenoidectomy and Laterofixation | 59.9 | - | 1.83 ± 0.32 | 36 | 9 | 4 | 91 |
| Sellars (13)  (1971) | RCaS | 2 (1 male) | Arytenoidectomy and Cordopexy | 53 | 12.00 ±11.31 | 0.17 ± 0.12 | 50 | 50 | 4 | 100 |
| Yagudin et al. (14)  (2012) | PCaS | 21 (0 male) | Plastic Cordotomy | 56 | 6.58 | 1.20 | 14 | 0 | 4 | 100 |
| Zealear et al. (15)  (2002) | PCaR | 1 (0 male) | Laryngeal Pacemaker and Botulinum-Toxin Injection | 63 | 1,5 | 2.33 | 100 | 0 | 4 | 100 |

Excluded small (< 3) subgroups. SC = severe complications, RCaS = retrospective case series, RCoS = retrospective cohort study, PCaS = prospective case series, PCoS = prospective cohort study, CEBM = oxford center of medicine score, SD = standard deviation, *no differentiation between female and male patients.

**References**

1. Anand V, Kumaran BR, Chenniappan S. Cordoplasty: a new technique for managing bilateral vocal cord paralysis and its comparison with posterior cordotomy and external procedure in a large study group. *Indian journal of otolaryngology and head and neck surgery*. 2015;67:40-6. doi:10.1007/s12070-014-0740-4

2. Bahre H. Die Technik der von Wittmaack angegebenen Operationsmethode zur Behebung der Kehlkopfstenose bei doppelseitiger Medianstellung der Stimmbänder. *Archiv für Ohren-, Nasen- und Kehlkopfheilkunde*. 1930;127:41-50. doi:10.1007/BF01586489

3. Billante CR, Zealear DL, Courey MS, Netterville JL. Effect of chronic electrical stimulation of laryngeal muscle on voice. *The Annals of otology, rhinology, and laryngology*. 2002;111:328-32. doi:10.1177/000348940211100408

4. Gadkaree SK, Gelbard A, Best SR, Akst LM, Brodsky M, Hillel AT. Outcomes in Bilateral Vocal Fold Immobility: A Retrospective Cohort Analysis. *Otolaryngology*. 2018;159:1020-7. doi:10.1177/0194599818800462

5. Gupta AK, Mann SB, Nagarkar N. Surgical management of bilateral immobile vocal folds and long-term follow-up. *The Journal of laryngology and otology*. 1997;111:474-7. doi:10.1017/s0022215100137685

6. Jackson C. Ventriculocordectomy: A new operation for the cure of goitrous paralytic laryngeal stenosis. *Archives of Surgery*. 1922;4:257-74. doi:10.1001/archsurg.1922.01110110003001

7. Langnickel R. An endolaryngeal method of vertico-lateral transposition of the vocal cord for bilateral abductor paralysis. *The Laryngoscope*. 1976;86:1020-8. doi:10.1288/00005537-197607000-00018

8. Müller AH, Hagen R, Pototschnig C, et al. Laryngeal pacing for bilateral vocal fold paralysis: Voice and respiratory aspects. *The Laryngoscope*. 2017;127:1838-44. doi:10.1002/lary.26428

9. Nawka T, Sittel C, Gugatschka M, et al. Permanent transoral surgery of bilateral vocal fold paralysis: A prospective multi-center trial. *The Laryngoscope*. 2015;125:1401-8. doi:10.1002/lary.25137

10. Pia F, Pisani P, Aluffi P. CO 2 laser posterior ventriculocordectomy for the treatment of bilateral vocal cord paralysis. *European Archives of Oto-Rhino-Laryngology*. 1999;256:403-406. doi:10.1007/s004050050175

11. Ptok M, Schönweiler R. Botulinum-Toxin-A-induzierte „Rebalanzierung“ bei beidseitiger Stimmlippenparese? *HNO*. 2001;49:548-52. doi:10.1007/s001060170080

12. Remsen K, Lawson W, Patel N, Biller HF. Laser lateralization for bilateral vocal cord abductor paralysis. *Otolaryngology*. 1985;93:645-9. doi:10.1177/019459988509300514

13. Sellars SL. Endolaryngeal surgery of bilateral vocal cord abductor paralysis. *Suid-Afrikaanse tydskrif vir geneeskunde*. 1971;45:1337-9.

14. Yagudin RK, Demenkov VR, Yagudin KF. Plastic cordotomy in the treatment of bilateral vocal fold immobility. *Head & neck*. 2012;34:1753-8. doi:10.1002/hed.22002

15. Zealear DL, Billante CR, Courey MS, Sant'Anna GD, Netterville JL. Electrically stimulated glottal opening combined with adductor muscle botox blockade restores both ventilation and voice in a patient with bilateral laryngeal paralysis. *The Annals of otology, rhinology and laryngology*. 2002;111:500-6. doi:10.1177/000348940211100605
