# Supplementary material for: Surgery for bilateral vocal fold paralysis: Systematic review and meta-analysis
Source: Front Surg. 2022 Jul 22;9:956338. doi: 10.3389/fsurg.2022.956338 (PMC9354550; doi:10.3389/fsurg.2022.956338)
Supplement: Supplementary file 2 [file Data_Sheet_1_v1.docx]

**Supplement figures**

**Surgery for Bilateral Vocal Fold Paralysis: Systematic Review and Meta-Analysis**

Kai Titulaer^1^, Peter Schlattmann, MD PhD^2^ Orlando Guntinas-Lichius, MD^1*^

^1^Department of Otorhinolaryngology, Jena University Hospital, Jena, Germany

^2^Department of Medical Statistics, Computer Sciences and Data Sciences, Jena University Hospital, Jena, Germany

**Supplement Figure 1**

**837 titles retrieved**

**645** articles eliminated
(not matching inclusion criteria)

**192** abstracts reviewed

**78** articles eliminated (study type, animal studies, same dataset, etc.)

**114** articles reviewed

**12** articles eliminated (subgroup size n < 3)

**102** articles selected, **2802** patients

**Supplement Figure 2**

**
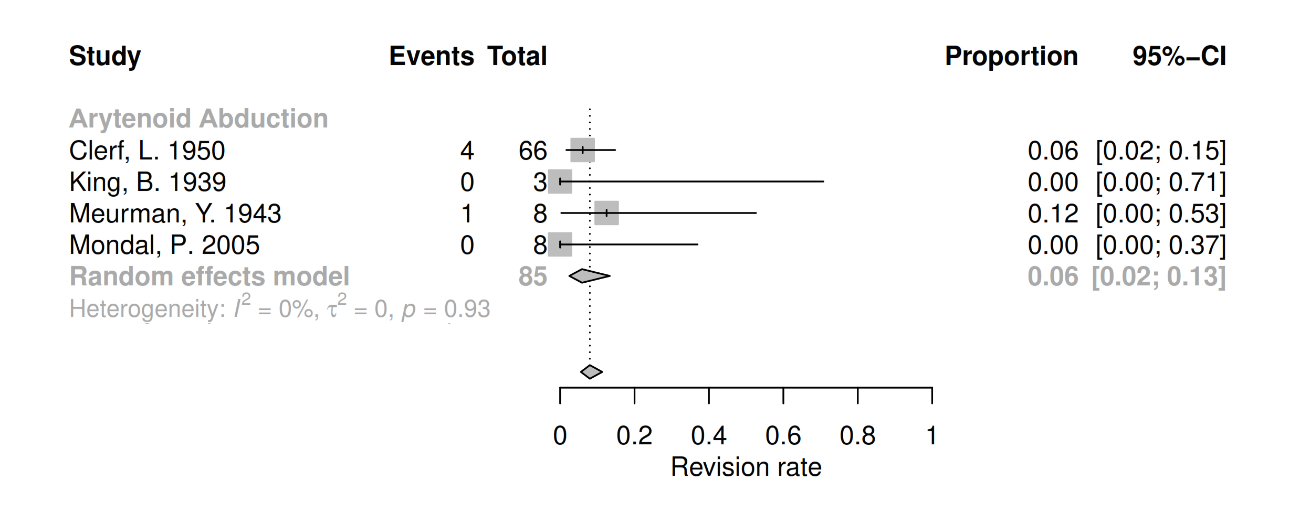
**

**Supplement Figure 3**

**
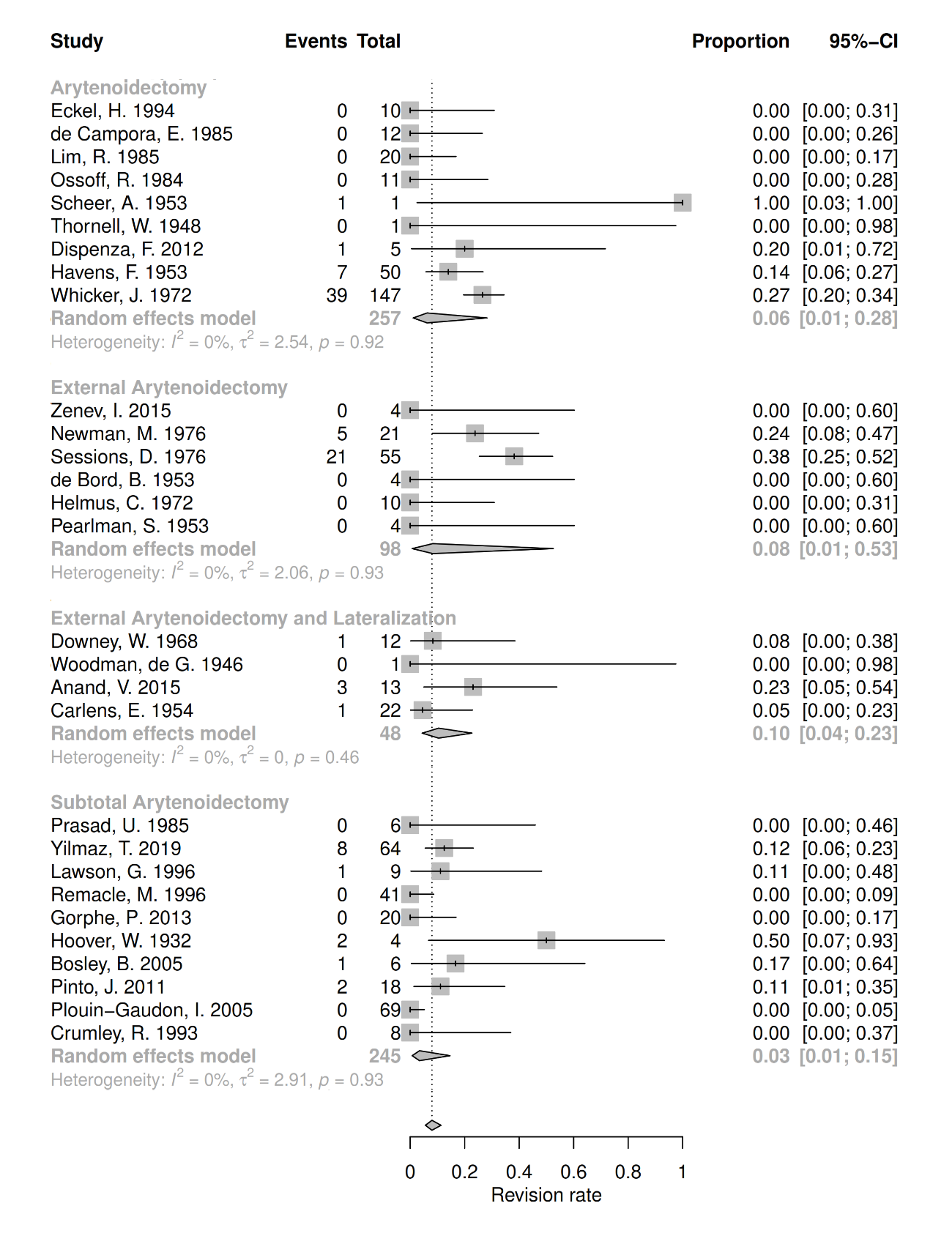
**

**Supplement Figure 4**

**
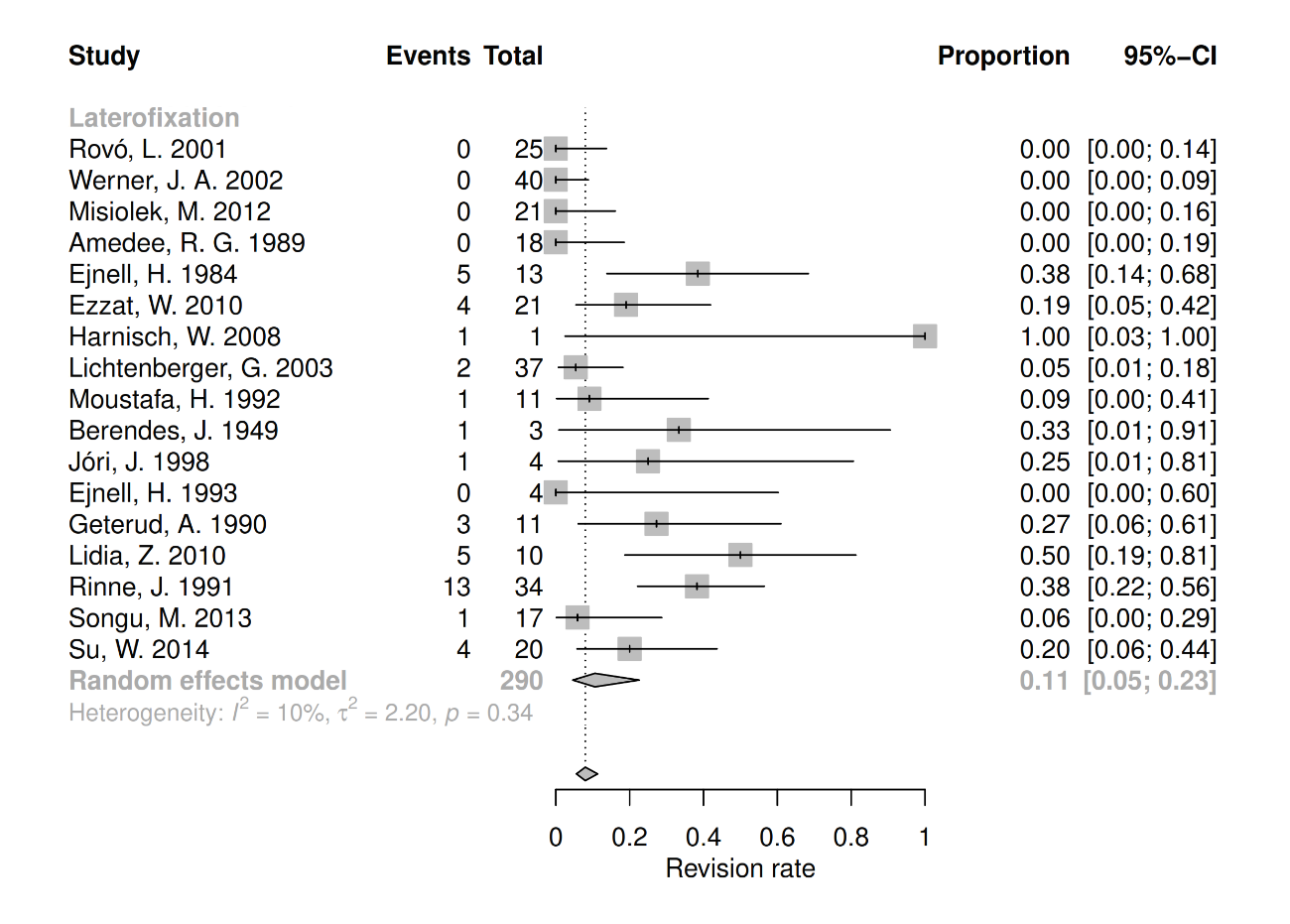
**

**Supplement Figure 5**

**
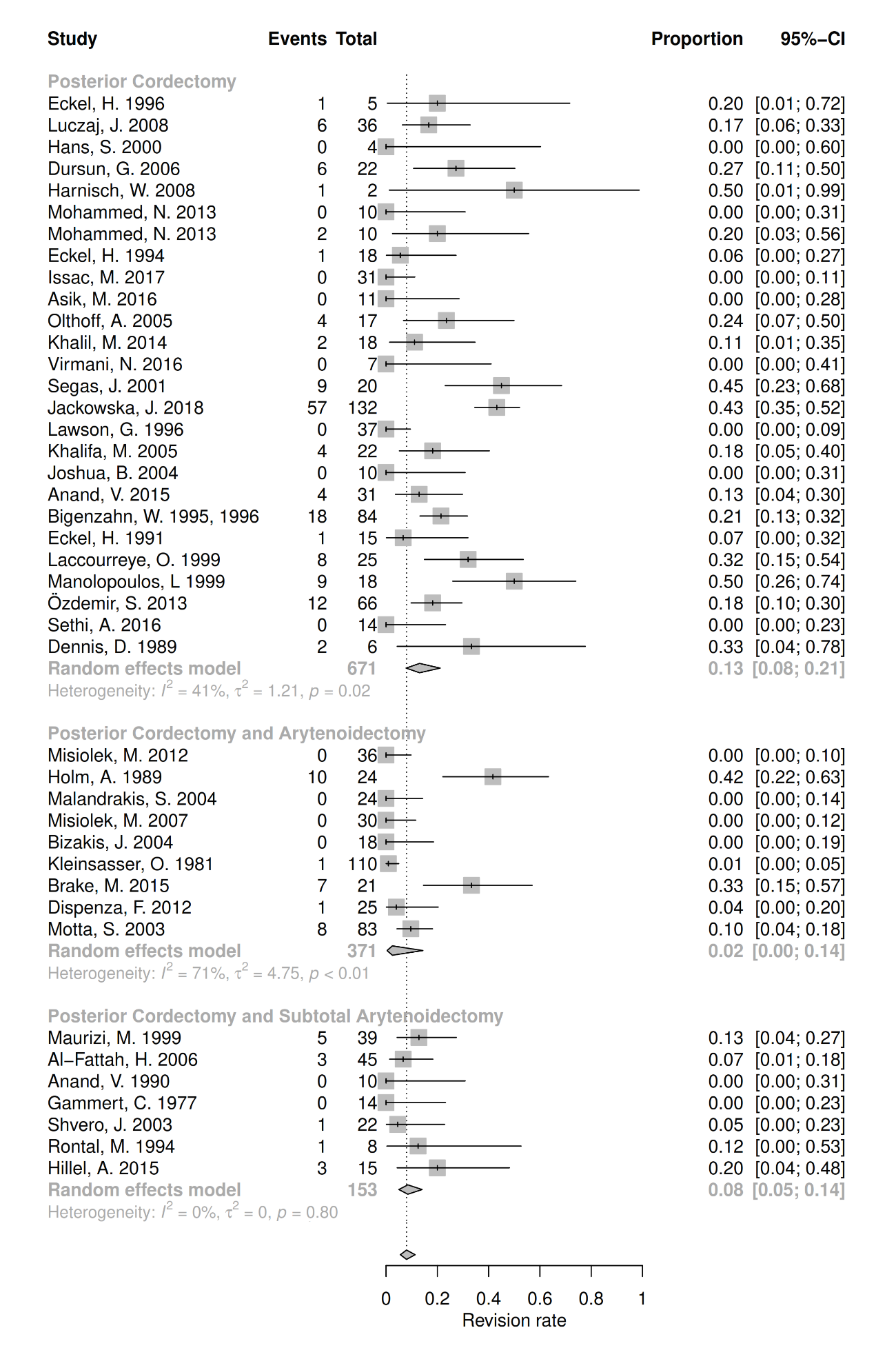
**

**Supplement Figure 6**

**
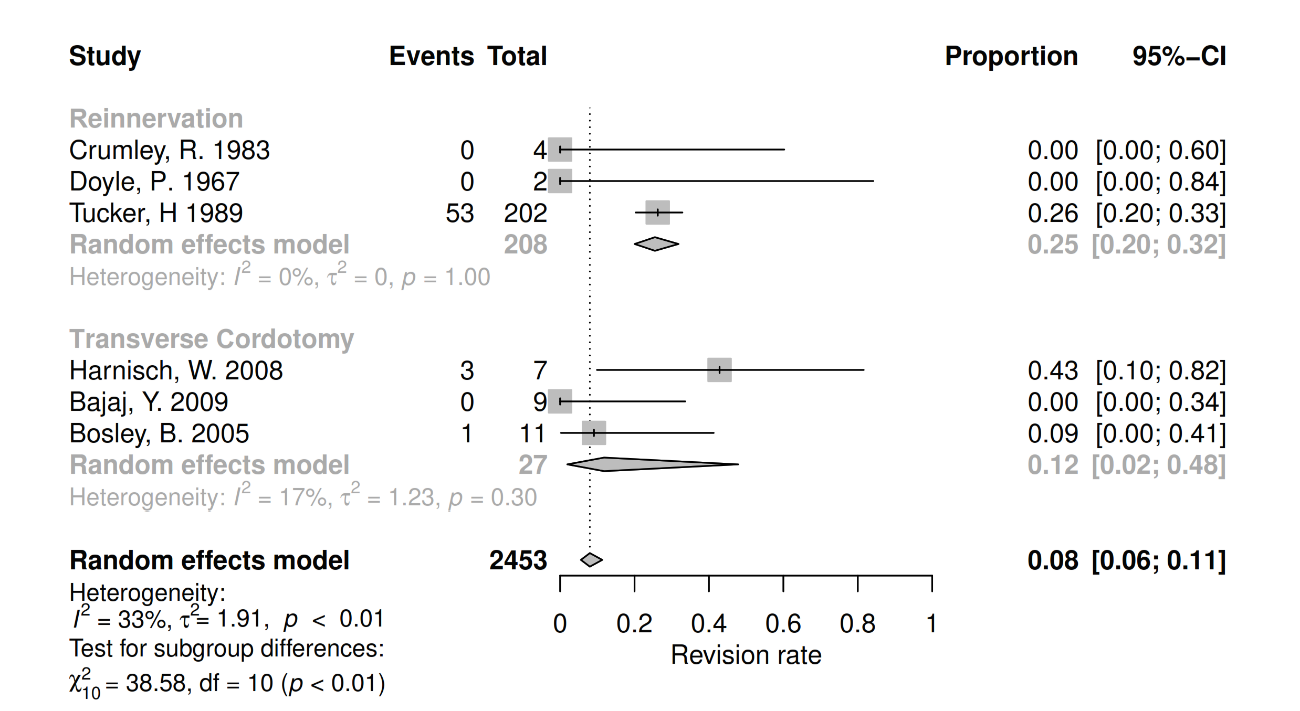
**
